# Supplementary material for: Strontium isoscape of sub-Saharan Africa allows tracing origins of victims of the transatlantic slave trade
Source: Nat Commun. 2024 Dec 30;15:10891. doi: 10.1038/s41467-024-55256-0 (PMC11685951; doi:10.1038/s41467-024-55256-0)
Supplement: Supplementary file 1 — Supplementary Information [file 41467_2024_55256_MOESM1_ESM.pdf]

Supplementary Materials for

Strontium isoscape of sub-Saharan Africa allows tracing origins of victims of the transatlantic slave trade

Wang et al.

Email: voelze@ucsc.edu

This PDF file includes:

Supplementary Notes 1 to 3
Supplementary Figures 1 to 6
Supplementary References

Supplementary Notes

Note 1. Strontium isotope biogeochemistry and strontium isoscapes

$^{87}Sr/^{86}Sr$ analysis has been widely used to reconstruct ancient and modern human and animal movement patterns in a wide range of fields including archaeology, ecology, forensic sciences, and food provenance studies[1]. Strontium has four naturally occurring stable isotopes: ^{84}Sr (0.56%), ^{86}Sr (9.87%), ^{87}Sr (7.04%), and ^{88}Sr (82.53%). Unlike the other three isotopes, ^{87}Sr is radiogenic because it is produced by radioactive β-decay of rubidium-87 (^{87}Rb) with a half-life of 48.8 billion years[2]. Therefore, rocks with different ages and distinctive elemental Rb/Sr ratios show large variation in $^{87}Sr/^{86}Sr$ composition, resulting in different geological units exhibiting different $^{87}Sr/^{86}Sr$ characteristics. For instance, old rocks composed of minerals with high K and Rb contents (e.g. old crystalline rocks such as granites or gneisses) will generally have high $^{87}Sr/^{86}Sr$ ratios, while young rocks rich in minerals with low K and Rb contents (e.g. basalts and limestone) are commonly characterized by low $^{87}Sr/^{86}Sr$ ratios[3,4]. Because of the long half-life decay of ^{87}Rb, $^{87}Sr/^{86}Sr$ ratios change very little on an archaeological time scale.

Strontium in rocks is released into water and soils by rock weathering, and becomes biologically available (bioavailable strontium) to be taken up by plants and later animals and humans through the food chain[4]. With similar ionic radius and chemical properties to calcium, strontium substitutes calcium in bioapatite tissues (e.g., teeth and bones) of humans and animals during tissue formation. During these processes, strontium has very minimal mass-dependent fractionation which is corrected during post-measurement data processing[1]. In the case of human teeth, $^{87}Sr/^{86}Sr$ ratios of tooth enamel which form during childhood and early adulthood and is highly resistant to post-mortem alteration, can reflect the $^{87}Sr/^{86}Sr$ signature of the geological location where an individual spent her childhood or early adulthood depending on the tooth analyzed[5]. Because enamel is not remodeled once fully mineralized, enamel $^{87}Sr/^{86}Sr$ ratios reflect those of the bedrock primarily inhabited by the individual during tooth mineralization[6]. By comparing individual enamel $^{87}Sr/^{86}Sr$ ratios with an established $^{87}Sr/^{86}Sr$ baseline we can identify non-locals within a sampled population.

While largely derived from the underlying bedrock geology, the isotopic compositions of bioavailable Sr and living organisms (e.g. plants and snail shells) can differ from the $^{87}Sr/^{86}Sr$ ratios of whole rocks and bulk soils[7,8]. This is because some factors can also potentially influence the biosphere $^{87}Sr/^{86}Sr$ in a given region, including differential mineral compositions and weathering rates of different rock types, topographical processes, as well as non-geological sources from atmospheric deposition (e.g., rainfall, dust, and coastal sea-spray) and anthropogenic pollution[9-11]. Thus, in practice, analyzing biosphere samples is more representative of local $^{87}Sr/^{86}Sr$ characteristics[8].

To assess the geological origins of archaeological material, knowledge of the bioavailable $^{87}Sr/^{86}Sr$ landscape (isoscape) of a given region is required. Recently, many

Sr isoscapes with varying scales and resolutions have been developed using different approaches for either local or large-scale regions in various parts of the world[11,12]. Baseline samples used in these studies include plants, snail shells, low-mobility archaeological/modern faunal remains (teeth and bones), soil leachates, and surface andshallow ground waters[13-15]. Researchers often use a combination of these baseline samples with different modeling methods to predict $^{87}Sr/^{86}Sr$ isoscapes[1,15,16]. The modeling methods that are commonly used in these studies include geostatistical spatial interpolation models (e.g., kriging and inverse distance weighting[16,17]), Bayesian models[18], and spatial aggregation methods[11,19]. More recently, random forest (RF) regression models based on the combination of environmental $^{87}Sr/^{86}Sr$ data and a range of environmental predictors were introduced into the mapping of large-scale Sr isoscapes[20,21], and greatly improved the predictive power of isoscapes. Overall, numerous isoscapes built by different methods can be found in many regions of the world, including Europe[11,16,19,20-22], Alaska[23], North America[24], part of South America[25], China[15], South Korea[26], north-east Australia[27], Israel[28], Turkey[29], New Zealand[30], part of Africa[31,32], Madagascar[1], and even globally[1]. However, the global Sr isoscape using the RF modeling method remains inaccurate in regions with sparse or limited sample data, particularly in Africa.

In this study, our sampling strategy includes plants (mostly modern plants and very few archaeological plants), soil leachates, snail shells, and teeth and bones of archaeological fauna (Supplementary Data 1). We combined these new data points with published environmental data, excluding water samples. Water samples are often obtained along the course of large drainages, and this type of sampling obscures local $^{87}Sr/^{86}Sr$ variation, as rivers often cross and mix isotopic signals from different lithological units[1].

Note 2. Geological background of sub-Saharan Africa

Sub-Saharan Africa is composed of several major Archean cratons (e.g., Kalahari [including Zimbabwe and Kaapvaal cratons], Congo, and West African cratons) and smaller cratonic fragments. The Late Neoproterozoic to earliest Paleozoic Pan-African orogeny led to the amalgamation of these cratons, and the final consolidation of the African continent[33]. Overall, the whole of the continent exposes crystalline Precambrian basement and the Phanerozoic cover rocks. The regional geology is summarized below, along with the geographic division of Africa according to the United Nations geoscheme for Africa[34].

2.1 West Africa

West Africa is mainly made of three major geological domains: the West African Craton, Benin-Nigeria Block, and Senegalo-Mauritanian Basin (Supplementary Fig. 4). The West African Craton comprises Archean nuclei (3.5-2.7 Ga) to the west, and Paleoproterozoic greenschist (2.35-2 Ga), volcano-sedimentary rocks and granitoids to the east[35]. The basement is largely covered by Neoproterozoic-Paleozoic sediments in the Taoudeni Basin of the central craton and the Volta Basin of the southwestern craton, respectively. The Benin-Nigeria Block consists mainly of Archean gneisses (3.7-2.9 Ga) and deformed Paleoproterozoic greenstone (2.2-2.0 Ga), which were intruded by Paleoproterozoic and Pan-African (870-520 Ma) granites[33]. The Senegalo-Mauritanian Basin, the largest basin on the northwest African Atlantic margin, is largely covered by the Cenozoic sediments.

2.2 Central Africa

Central Africa is dominated by the Congo Craton, which is largely covered by thick Mesozoic-Cenozoic sediments and exposes the ancient basement and orogenic belts mainly at the edges. Archean bedrock is primarily exposed in the Gabon-Kamerun Shield in the northwest, the Angola Block in the southwest, Kasai Shield in the southeast, and the NE Congo Block in the northeast (Supplementary Fig. 4). More specifically, the Gabon-Kamerun Shield in the northwest and the Kasai Shield in the southeast mainly consist of Archean plutonic rocks, with remnants of older rocks[33]. The core of the Angola Block is represented by widespread felsic Eburnean (~2 Ga) plutonic and metamorphic rocks[36]. The NE Congo Block contains Archean granitic gneisses and greenstone belts, as well as Paleoproterozoic metamorphosed granites.

The orogenic belts are mainly located along the northern and western margins of the Congo Craton (Supplementary Fig. 4). The Central African Fold Belt extends nearly in a west-east direction, with Meso-Neoproterozoic volcano-sedimentary rocks overlying the Paleoproterozoic (2.0 Ga) basement[37]. The West Congo Belt, located on the western

margin of the Congo Craton, consists of unmetamorphosed Neoproterozoic strata in the east, Pan-African Belts in the middle and reworked Precambrian basements in the west[38].

2.3 Southern Africa

About two-thirds of South Africa is covered by rocks of the Upper Carboniferous to Lower Jurassic Karoo Supergroup, primarily comprising continental clastic sediments in the Main Karoo Basin throughout the country and Jurassic Karoo basalts in the east of the country (Supplementary Fig. 4)[39]. The Kaapvaal Craton in northeastern South Africa consists predominantly of Paleoarchean to Rhyacian granitoids and gneisses, interspersed with greenstone belts and overlain by Neoarchean volcanic and sedimentary sequences[40]. The Namaqua Belt in northwest South Africa is composed of Paleoproterozoic to Mesoproterozoic deformed and metamorphosed granitic gneisses and supracrustal rocks[41]. The Cape Fold Belt along the southern and western coast is composed of Paleozoic sedimentary and volcano-sedimentary rocks, as well as the Paleozoic granite and Neoproterozoic-Paleozoic metasedimentary rocks of the Pan-African orogeny[42]. In the Kalahari Basin, which stretches ~2200 km in the hinterland from the Congo to South Africa, sediments up to 450m-thick have been deposited since the Late Cretaceous[43].

2.4 East Africa

East Africa has a very complex geological setting. Several major Archean cratons (e.g., Zimbabwe Craton, Tanzania Craton, and Ugandan Craton) and Paleoproterozoic microcontinental blocks (Bangweulu Block) are flanked by younger orogenic belts (e.g., Irumide Belt, East African Orogenic Zone, Kibaran Belt, Zambezi Belts, and Lufilian Belts) (Supplementary Fig. 4). The Zimbabwe Craton in southern East Africa mainly consists of Archean (3.5-2.8 Ga) granitic gneiss, partly overlain by younger (2.8-2.6 Ga) and Paleoproterozoic volcanic and sedimentary rocks, as well as granitoids (Supplementary Fig. 4)[44,45]. The Bangweulu Block in central-southern East Africa is primarily composed of Paleoproterozoic plutonic and volcano-sedimentary rocks. The Tanzania Craton, located in the central part of East Africa, is mainly composed of Archean granitoids and greenstone belts[46], with some Mesozoic to Cenozoic volcanic rocks in the northeast. In central-northern East Africa, most regions of Uganda expose Archean gneisses and granites, which underwent intense Paleoproterozoic (2.9 and 2.55 Ga) and Neoproterozoic (ca. 650 Ma, Pan-African orogeny) metamorphism (Supplementary Fig. 4)[47,48]. The Irumide Belt, separating the Zimbabwe Craton to the south and Bangweulu Block to the north, is mainly overlain by Mesoproterozoic plutonic and volcano-sedimentary rocks, which experienced widespread metamorphism and granitoid magmatism at ~1.1-1.0 Ga[49,50]. The Lufilian Belt mainly consists of Neoproterozoic rift-related volcanic and sedimentary sequences deposited on the passive margins of the Congo and Kalahari cratons[50]. The Kibaran Belt,

located between the Tanzania and Congo cratons, consists of a Paleoproterozoic volcano-sedimentary sequence in the southeastern part and Mesoproterozoic sediments, which were intruded by younger (1.38-1.37 Ga) granites[51,52]. The East African Orogenic Zone in the northeastern parts of East Africa represents a wide suture zone between East Gondwana and the various plates of West Gondwana during the Neoproterozoic (800-580 Ma)[53,54]. Many regions within the East African Orogenic Zone (e.g., Kenya, Ethiopia, northeastern Tanzania, southeastern Sudan, and Eritrea) are widely covered by Mesozoic-Cenozoic volcanic rocks and sediments (Supplementary Fig. 4). For example, in Ethiopia, the basement is overlain by rift-related rhyolitic and basaltic lavas that erupted at ∼30 Ma[55].

2.5 Southeastern North Africa

The East Saharan Metacraton occupies most of North Africa. In southeastern North Africa, this metacraton is largely covered by Phanerozoic sedimentary sequences, with the exposed areas primarily consisting of Neoproterozoic medium- to high-grade plutonic gneisses and migmatites[37] (Supplementary Fig. 4).

Note 3. $^{87}Sr/^{86}Sr$ variability in sub-Saharan Africa

As one of the most geologically diverse regions in the world, sub-Saharan Africa exhibits significant heterogeneity in $^{87}Sr/^{86}Sr$ ratios across large spatial scales. The $^{87}Sr/^{86}Sr$ ratios for the entire sub-Saharan Africa dataset range from 0.70381 to 0.87810, with a mean of 0.71774 ± 0.01229 (1σ), reflecting the continent's diverse geological characteristics. The distribution of these data is markedly asymmetric (kurtosis = -268.2, skewness = 3.1, Supplementary Fig. 5) and non-normally distributed (Shapiro–Wilk test: $p < 0.05$; Supplementary Fig. 5). Specifically, 25% of the data have low radiogenic compositions (0.703–0.710), 45% fall between 0.710 and 0.720, 29% lie between 0.720 and 0.760, and the remaining 1% have extremely high ratios (0.760–0.880).

Spatially, the $^{87}Sr/^{86}Sr$ ratios exhibit distinct patterns across different geological units in Africa (Supplementary Fig. 6). In West Africa, the ratios are relatively homogeneous and intermediate compared to other regions. The lowest ratios in this area are found in the Senegalo-Mauritanian Basin (0.712 ± 0.002, 1σ, n = 15) in present-day Senegal, The Gambia, and Guinea-Bissau, as well as in other coastal basins along the Atlantic Ocean (e.g., southern Benin and Nigeria [0.712 ± 0.003, n = 11]). Slightly elevated ratios are observed in the Rockellides Belt (0.714 ± 0.001, n = 28; present-day western Guinea, eastern Guinea-Bissau, and southeastern Senegal) and the Benin-Nigeria Block (0.716 ± 0.006, n = 18; present-day Benin and Nigeria). The highest $^{87}Sr/^{86}Sr$ ratios (~0.720–0.740) in West Africa are primarily found in the Man-Leo Shield, located in the southern part of the West African Craton (0.719 ± 0.009, n = 196; present-day Côte d'Ivoire, Liberia, and Sierra Leone), with a few samples also found in the southwestern Taoudeni Basin, near the West African Craton (southwestern Mali). Extremely high ratios (~0.740–0.770) are observed only in the Man-Leo Shield.

Central Africa exhibits a wide range of isotopic ratios, spanning from 0.703 to 0.768 (Supplementary Fig. 6). The lowest $^{87}Sr/^{86}Sr$ ratios are found in the Central Africa Fold Belt (0.715 ± 0.003, n = 47; present-day Cameroon, Central African Republic, and the border regions between Nigeria and Cameroon). Comparatively higher ratios are observed in the Congo Basin, NE Congo Belt, and Lindian Domain (0.721 ± 0.04, n = 61; present-day Democratic Republic of the Congo), the West Congo Belt (0.723 ± 0.07, n = 26; present-day eastern Gabon, southwestern Republic of the Congo, western Democratic Republic of the Congo, and northern Angola), and the Gabon-Kamerun Shield (0.723 ± 0.08, n = 27; present-day Gabon). The most radiogenic $^{87}Sr/^{86}Sr$ ratios are found in the Angola Block in south-central Angola (0.742 ± 0.008, n = 26).

The $^{87}Sr/^{86}Sr$ ratios in East Africa and southeastern North Africa exhibit complex variation. The lowest ratios are widely distributed across the East African Orogenic Zone (0.707 ± 0.003, n = 345; present-day Ethiopia, Kenya, Somalia, eastern Sudan, and northeastern Tanzania). Slightly elevated ratios are commonly found in the Tanzania Craton (0.711 ± 0.003, n = 59; present-day Tanzania) and Ugandan Craton (0.714 ± 0.006,

n = 152; present-day Uganda). In contrast, the $^{87}Sr/^{86}Sr$ ratios in the Zimbabwe Craton are highly variable and more radiogenic (0.754 ± 0.041, n = 25; present-day Zimbabwe), with the highest ratio reaching 0.8781.

Our data from southern Africa are primarily from South Africa, with only a few samples from Namibia (0.722 ± 0.004, n = 8) and Botswana (0.718 ± 0.007, n = 4). The $^{87}Sr/^{86}Sr$ variation in South Africa exhibits a strong north-south gradient. The highest ratios are found in the Kaapvaal Craton (0.734 ± 0.011, n = 264) in northern South Africa, with a few samples reaching 0.760–0.790. The lowest ratios occur in the Karoo Basalt regions of east-central South Africa (0.709 ± 0.002, n = 16). The Cape Fold Belt in southern South Africa has slightly higher ratios (0.713 ± 0.04, n = 405). The Main Karoo Basin, Kalahari Basin, and Namaqua Belt show elevated compositions, with $^{87}Sr/^{86}Sr$ ranges of 0.720 ± 0.012 (n = 242), 0.720 ± 0.002 (n = 6), and 0.721 ± 0.001 (n = 27), respectively.

Overall, the $^{87}Sr/^{86}Sr$ ratios in sub-Saharan Africa span a broad range, reflecting regional patterns largely driven by the diverse geological conditions of each tectonic block. Africa generally has higher $^{87}Sr/^{86}Sr$ ratios than any other continent due to its largely crystalline Precambrian basement. The lowest ratios (< 0.709) are primarily found in: 1) regions with young mafic/ultramafic volcanic rocks, such as the East African Orogenic Zone, where Cenozoic rhyolitic and basaltic rocks are prevalent; 2) southeast South Africa, where Karoo basalts are widespread; and 3) areas in northwestern Cameroon covered by Mesozoic volcanic rocks. Radiogenic ratios (> 0.730) are restricted to regions dominated by Archean bedrock, including the southern Western Africa Craton (Man-Leo Shield), the southwestern Congo Craton (Angola Block), and the Kalahari Craton (Kaapvaal and Zimbabwe Cratons), likely reflecting the weathering of Archean granitoids and metamorphic rocks. High $^{87}Sr/^{86}Sr$ regions include present-day Angola, Zimbabwe, Zambia, western Tanzania, northern South Africa, and southern West African countries. The most common $^{87}Sr/^{86}Sr$ ratios (0.710–0.730) are widely distributed across various geological units, including coastal basins with upper Cretaceous to Cenozoic sediments (0.709–0.720), Neoproterozoic to early Paleozoic (Pan-African) orogenic belts (e.g., Cape Fold Belt and Central African Fold Belt), the Taoudeni Basin, and regions with Archean-Paleoproterozoic basements (e.g., Ugandan Craton, Benin-Nigeria Block) and the Main Karoo Basin, dominated by thick Paleozoic-Mesozoic clastic sediments.

Supplementary Figures

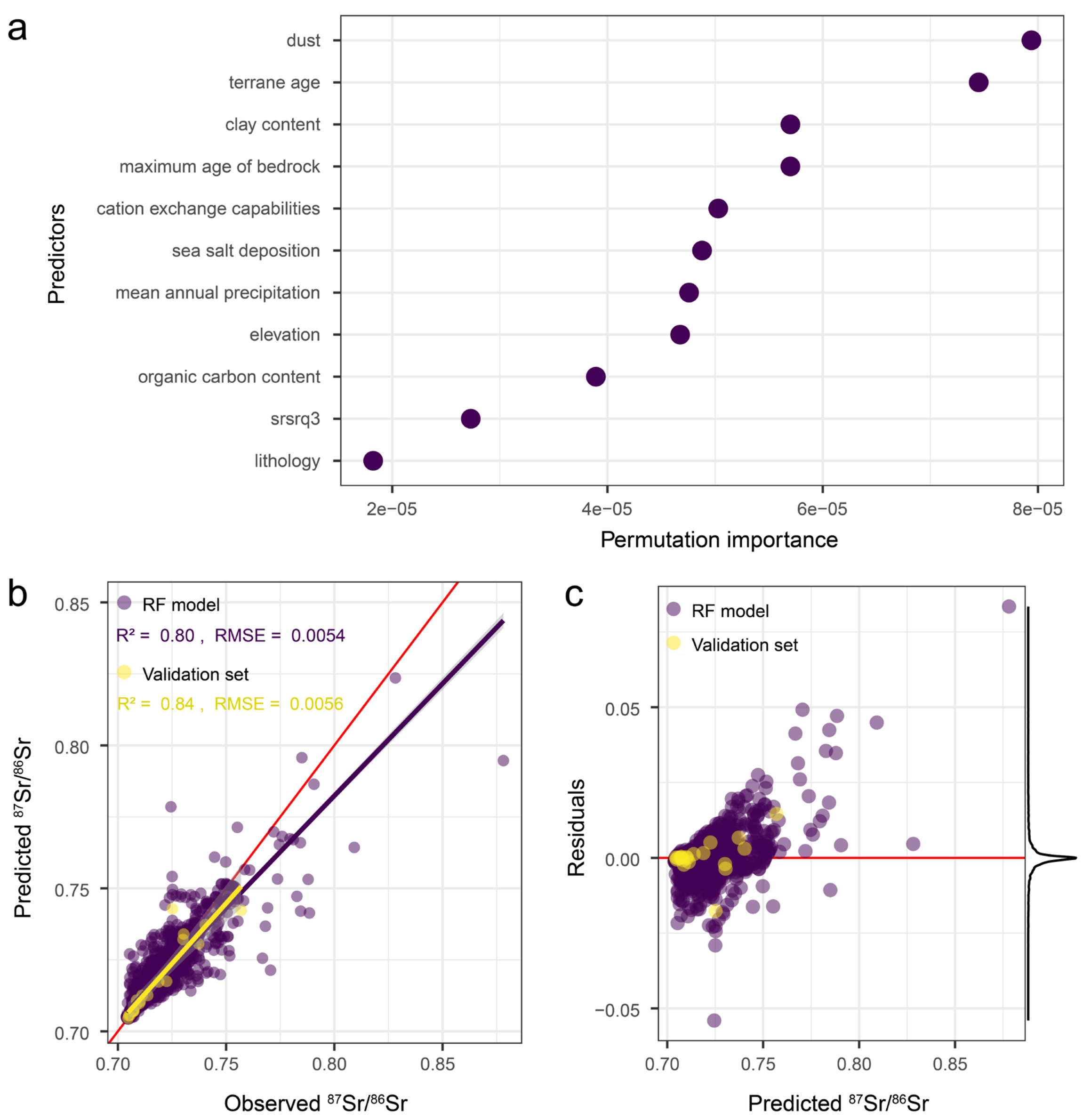

Supplementary Figure 1. Modeling results. **a** Permutation importance of the predictors in the random forest model. **b** Predicted versus observed bioavailable $^{87}Sr/^{86}Sr$ ratios. Data points used for model training and cross-validation are shown in purple, while the 20 validation samples are in yellow. RMSE (root-mean-square error) indicates model accuracy. **c** Model residuals versus predicted bioavailable $^{87}Sr/^{86}Sr$ ratios, with the density plot of residuals on the right. Results from the random forest model are shown in purple, and the validation set is in yellow.

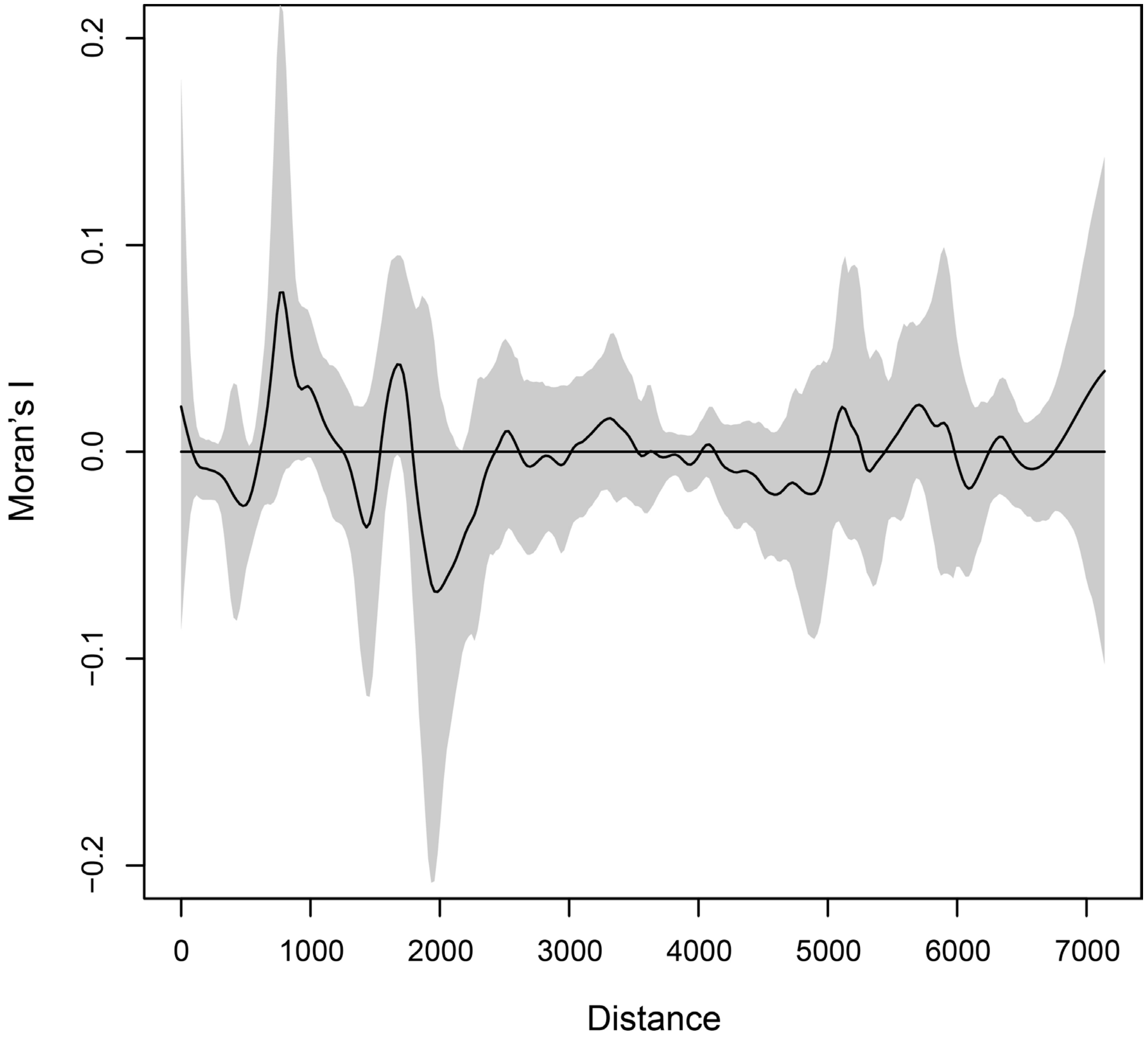

Supplementary Figure 2. Cross-correlogram showing the cross-correlation at different distances (km) between the model residuals and $^{87}Sr/^{86}Sr$ ratios. The generally low Moran's I values indicate that proximity (short distance) does not increase the correlation of residuals, suggesting the absence of spatial autocorrelation in the residuals. Additionally, the confidence interval (95%, grey shading) consistently includes zero, further supporting the absence of auto-correlation.

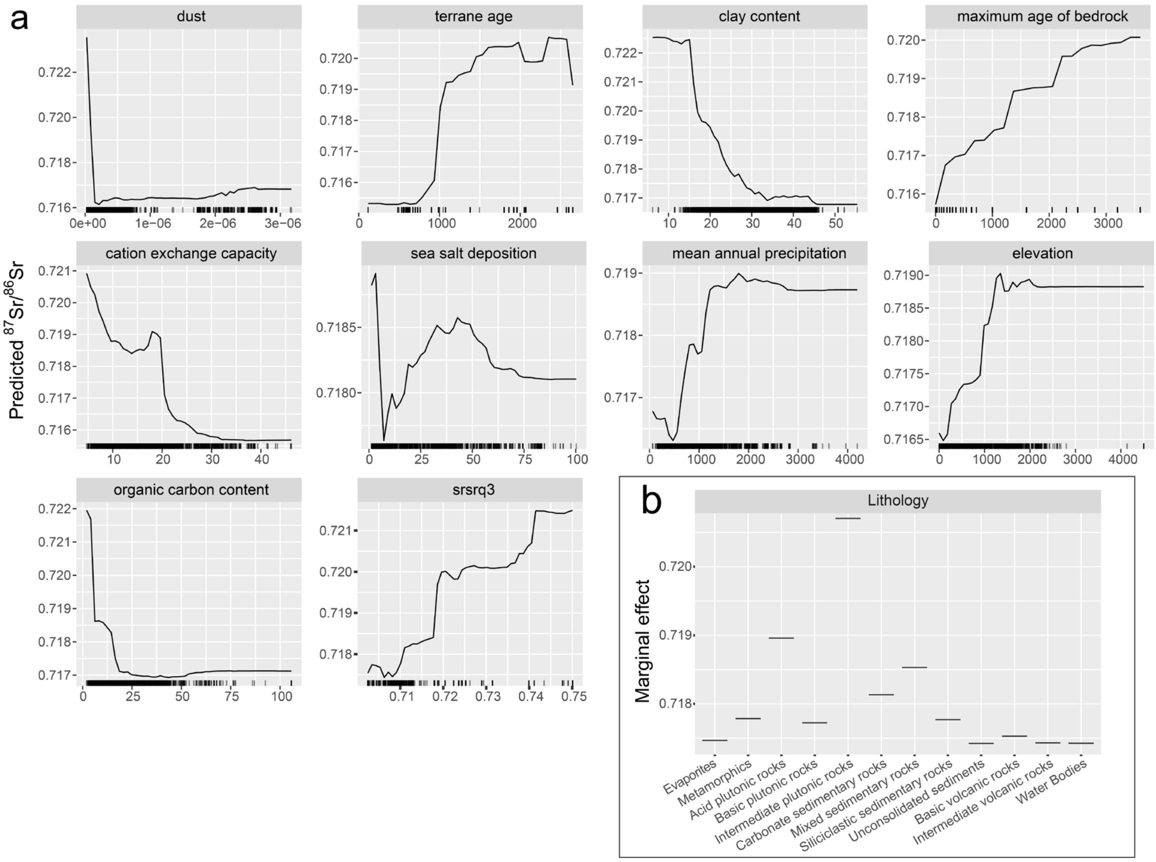

Supplementary Figure 3. Partial dependence plot between $^{87}Sr/^{86}Sr$ ratios and selected predictors. **a** Partial dependence plot between $^{87}Sr/^{86}Sr$ ratios and 10 selected predictors with continuous data. **b** Partial dependence plot between $^{87}Sr/^{86}Sr$ ratios and the categorical predictor (lithology).

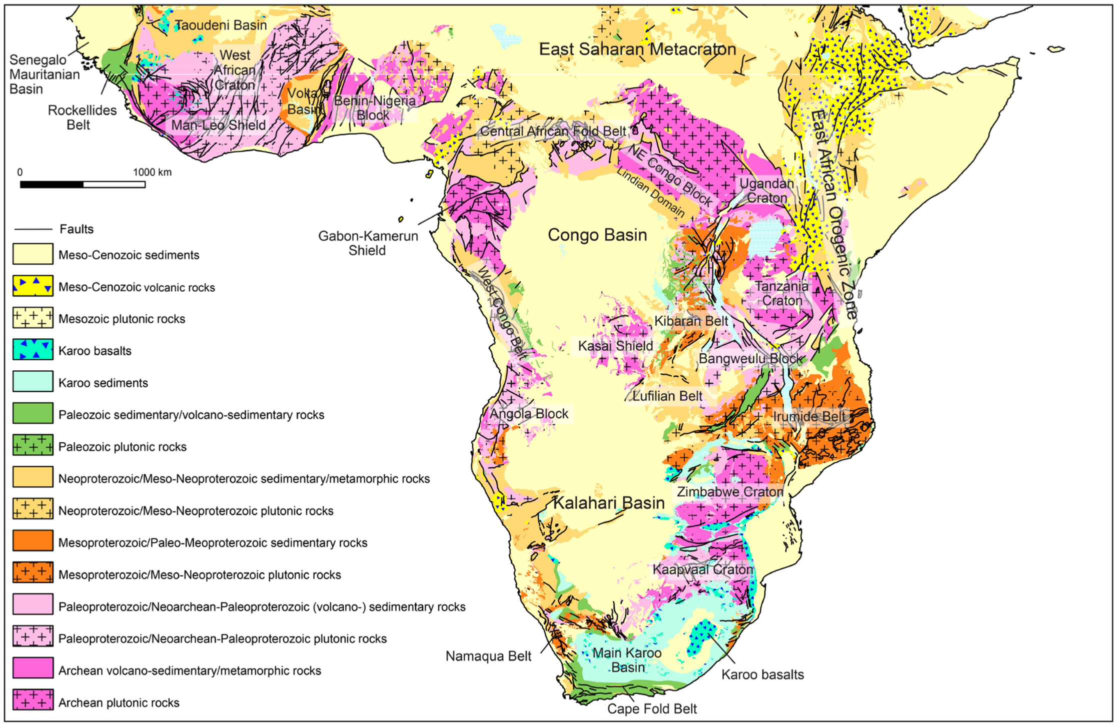

Supplementary Figure 4. Simplified geological map and major geological units of sub-Saharan Africa modified after refs. [33,56,57].

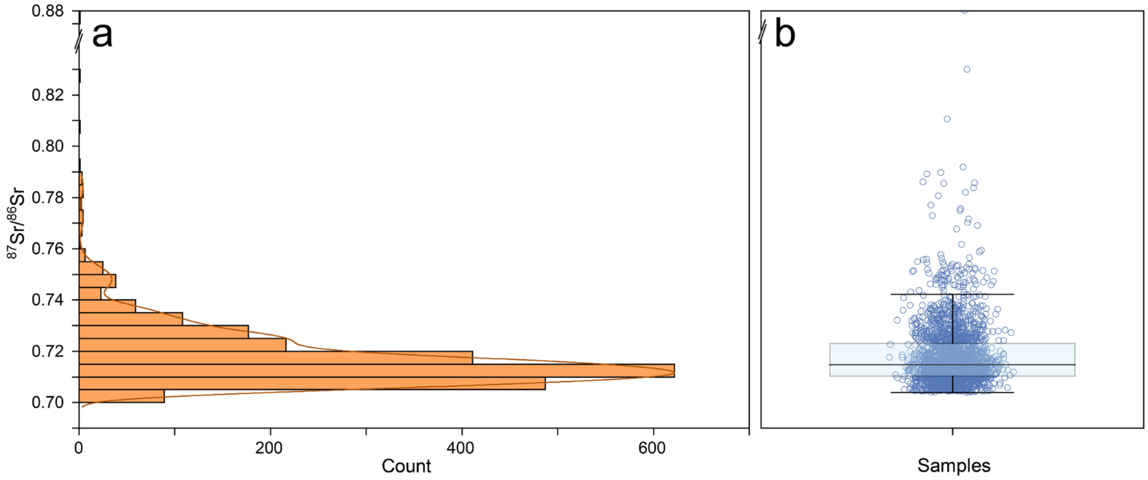

Supplementary Figure 5. Distribution of $^{87}Sr/^{86}Sr$ variability. **a** Histogram of $^{87}Sr/^{86}Sr$ ratios. The red curve shows the kernel density estimation of the data. **b** Box plot of $^{87}Sr/^{86}Sr$ data. The box represents the interquartile range (IQR) from the 25th to the 75th percentile, with a line indicating the median. Whiskers extend to the minimum and maximum values within 1.5 times the IQR, while outliers are data points beyond this range.

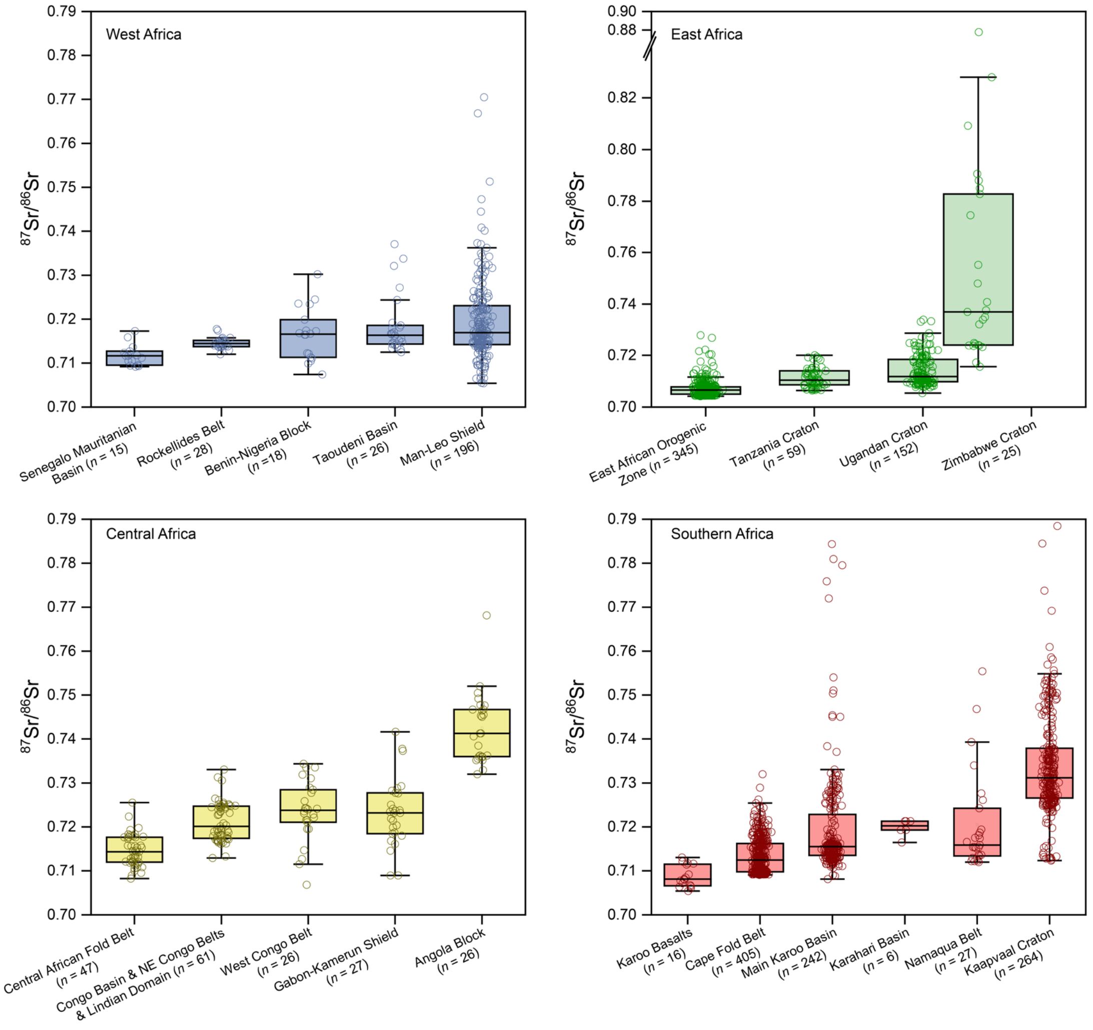

Supplementary Figure 6. Box plots of $^{87}Sr/^{86}Sr$ data for tectonic blocks and orogenic belts across Africa. The box represents the interquartile range (IQR) from the 25th to the 75th percentile, with a line indicating the median. Whiskers extend to the minimum and maximum values within 1.5 times the IQR, while outliers are data points beyond this range.

Supplementary References

1. Bataille, C. P. et al. Advances in global bioavailable strontium isoscapes. *Palaeogeogr. Palaeoclimatol. Palaeoecol.* **555**, 109849 (2020).
2. Faure, G. & Mensing, T. M. *Isotopes: Principles and Applications*. (Wiley, 2005).
3. Capo, R. C. et al. Strontium isotopes as tracers of ecosystem processes: theory and methods. *Geoderma* **82**, 197-225 (1998).
4. Bentley, R. A. Strontium isotopes from the earth to the archaeological skeleton: a review. *J. Archaeol. Method Theory* **13**, 135-187 (2006).
5. Slovak, N. M. & Paytan, A. in: *Handbook of Environmental Isotope Geochemistry: Vol I* (ed Baskaran, M.) 743-768 (Springer, 2012).
6. Copeland, S. R. et al. Strontium isotope investigation of ungulate movement patterns on the Pleistocene Paleo-Agulhas Plain of the Greater Cape Floristic Region, South Africa. *Quat. Sci. Rev.* **141**, 65-84 (2016).
7. Sillen, A. et al. $^{87}Sr/^{86}Sr$ ratios in modern and fossil food-webs of the Sterkfontein Valley: implications for early hominid habitat preference. *Geochim. Cosmochim. Acta* **62**, 2463-2473 (1998).
8. Price, T. D. et al. The characterization of biologically available strontium isotope ratios for the study of prehistoric migration. *Archaeometry* **44**, 117-135 (2002).
9. Thomsen, E. & Andreasen, R. Agricultural lime disturbs natural strontium isotope variations: Implications for provenance and migration studies. *Sci. Adv.* **5**, eaav8083 (2019).
10. Serna, A. et al. Implications for paleomobility studies of the effects of quaternary volcanism on bioavailable strontium: A test case in North Patagonia (Argentina). *J. Archaeol. Sci.* **121**, 105198 (2020).
11. Evans, J. A. et al. Spatial variations in biosphere $^{87}Sr/^{86}Sr$ in Britain. *J. Geol. Soc.* **167**, 1-4 (2010).
12. Beard, B. L. & Johnson, C. M. Strontium isotope composition of skeletal material can determine the birth place and geographic mobility of humans and animals. *J. Forensic Sci.* **45**, 1049-1061 (2000).
13. Hodell, D. A. et al. Spatial variation of strontium isotopes ($^{87}Sr/^{86}Sr$) in the Maya region: a tool for tracking ancient human migration. *J. Archaeol. Sci.* **31**, 585-601 (2004).
14. Britton, K. et al. Sampling plants and malacofauna in $^{87}Sr/^{86}Sr$ bioavailability studies: Implications for isoscape mapping and reconstructing of past mobility patterns. *Front. Ecol. Evol.* **8**, 579473 (2020).
15. Wang, X. & Tang, Z. The first large-scale bioavailable Sr isotope map of China and its implication for provenance studies. *Earth-Sci. Rev.* **210**, 103353 (2020).
16. Willmes, M. et al. Mapping of bioavailable strontium isotope ratios in France for archaeological provenance studies. *Appl. Geochem.* **90**, 75-86 (2018).
17. Emery, M. V. et al. Mapping the origins of Imperial Roman workers (1st–4th century CE) at Vagnari, Southern Italy, using $^{87}Sr/^{86}Sr$ and $\delta^{18}O$ variability. *Am. J. Phys. Anthropol.* **166**, 837-850 (2018).
18. Wang, X. et al. The circulation of ancient animal resources across the Yellow River basin: a preliminary Bayesian re-evaluation of Sr isotope data from the Early Neolithic to the Western Zhou Dynasty. *Front. Ecol. Evol.* **9**, 583301 (2021).
19. Snoeck, C. et al. Towards a biologically available strontium isotope baseline for Ireland. *Sci. Total Environ.* **712**, 136248 (2020).
20. Bataille, C. P. et al. A bioavailable strontium isoscape for Western Europe: A machine learning approach. *PLOS ONE* **13**, e0197386 (2018).
21. Hoogewerff, J. A. et al. Bioavailable $^{87}Sr/^{86}Sr$ in European soils: A baseline for provenancing studies. *Sci. Total Environ.* **672**, 1033-1044 (2019).
22. James, H. F. et al. A large-scale environmental strontium isotope baseline map of Portugal for

archaeological and paleoecological provenance studies. *J. Archaeol. Sci.* **142**, 105595 (2022).

23. Funck, J. et al. A bio-available strontium isoscape for eastern Beringia: a tool for tracking landscape use of Pleistocene megafauna. *J. Quat. Sci.* **36**, 76-90 (2021).
24. Reich, M. S. et al. Continuous-surface geographic assignment of migratory animals using strontium isotopes: A case study with monarch butterflies. *Methods Ecol. Evol.* **12**, 2445-2457 (2021).
25. Scaffidi, B. K. & Knudson, K. J. An archaeological strontium isoscape for the prehistoric Andes: Understanding population mobility through a geostatistical meta-analysis of archaeological $^{87}Sr/^{86}Sr$ values from humans, animals, and artifacts. *J. Archaeol. Sci.* **117**, 105121 (2020).
26. Shin, W. J. et al. Spatial distributions of strontium isotope ratios in human hair and tap water from South Korea. *Sci. Total. Environ.* **806**, 151352 (2022).
27. Adams, S. et al. A strontium isoscape of north-east Australia for human provenance and repatriation. *Geoarchaeology* **34(3)**, 231-251 (2019).
28. Moffat, I. et al. Bioavailable soil and rock strontium isotope data from Israel. *Earth Syst. Sci. Data* **12**, 3641-3652 (2020).
29. Wong, M. et al. A bioavailable baseline strontium isotope map of southwestern Turkey for mobility studies. *J. Archaeol. Sci. Rep.* **37**, 102922 (2021).
30. Kramer, R. T. et al. A bioavailable strontium ($^{87}Sr/^{86}Sr$) isoscape for Aotearoa New Zealand: Implications for food forensics and biosecurity. *PLOS ONE* **17**, e0264458 (2022).
31. Janzen, A. et al. Spatial variation in bioavailable strontium isotope ratios ($^{87}Sr/^{86}Sr$) in Kenya and northern Tanzania: Implications for ecology, paleoanthropology, and archaeology. *Palaeogeogr. Palaeoclimatol. Palaeoecol.* **560**, 109957 (2020).
32. Wang, X. et al. A bioavailable strontium isoscape of Angola with implications for the archaeology of the transatlantic slave trade. *J. Archaeol. Sci.* **154**, 105775 (2023).
33. Begg, G. C. et al. The lithospheric architecture of Africa: Seismic tomography, mantle petrology, and tectonic evolution. *Geosphere* **5**, 23-50 (2009).
34. United Nations Statistics Division, Methodology—standard country or area codes for statistical use (M49). https://unstats.un.org/unsd/methodology/m49/#geo-regions (1999). (last accessed January 28, 2024).
35. Brahimi, S. et al. The Tuareg shield terranes revisited and extended towards the northern Gondwana margin: Magnetic and gravimetric constraints. *Earth-Sci. Rev.* **185**, 572-599 (2018).
36. De Carvalho, H. et al. Geochronological review of the Precambrian in western Angola: links with Brazil. *J. Afr. Earth Sci.* **31**, 383-402 (2000).
37. Iizuka, T. et al. Evolution of the African continental crust as recorded by U–Pb, Lu–Hf and O isotopes in detrital zircons from modern rivers. *Geochim. Cosmochim. Acta* **107**, 96-120 (2013).
38. Dinis, P. et al. Climatic zonation and weathering control on sediment composition (Angola). *Chem. Geol.* **467**, 110-121 (2017).
39. Schlüter, T. *Geological Atlas of Africa*. (Springer, 2008).
40. Garzanti, E. et al. Provenance of Kalahari Sand: Paleoweathering and recycling in a linked fluvial-aeolian system. *Earth-Sci. Rev.* **224**, 103867 (2022).
41. Macey, P. H. et al. Implications of the distribution, age and origins of the granites of the Mesoproterozoic Spektakel Suite for the timing of the Namaqua Orogeny in the Bushmanland Subprovince of the Namaqua-Natal Metamorphic Province, South Africa. *Precambrian Res.* **312**, 68-98 (2018).
42. Blewett, S. C. & Phillips, D. in: *Origin and evolution of the cape mountains and Karoo Basin* (eds Linol, B. & de Wit Maarten, J.) 45-56 (Springer-Berlin, 2016).
43. Haddon, I. G. & McCarthy, T. S. The Mesozoic-Cenozoic interior sag basins of Central Africa: The

Late-Cretaceous–Cenozoic Kalahari and Okavango basins. *J. Afr. Earth Sci.* **43**, 316-333 (2005).

44. Kusky, T. M. Tectonic setting and terrane accretion of the Archean Zimbabwe craton. *Geology* **26**, 163-166 (1998).

45. Jelsma, H. A. & Dirks, P. H. G. M. Neoarchaean tectonic evolution of the Zimbabwe Craton. *Geol. Soc. London* **199**, 183-211 (2002).

46. De Waele, B. et al. Palaeoproterozoic to Neoproterozoic growth and evolution of the eastern Congo Craton: Its role in the Rodinia puzzle. *Precambrian Res.* **160**, 127-141 (2008).

47. Leggo Peter, J. A geochronological study of the basement complex of Uganda. *J. Geol. Soc.* **130**, 263-276 (1974).

48. Gabert, G. Lithostratigraphic and tectonic setting of gold mineralization in the Archean cratons of Tanzania and Uganda, East Africa. *Precambrian Res.* **46**, 59-69 (1990).

49. De Waele, B. & Fitzsimons, I. C. W. The nature and timing of Palaeoproterozoic sedimentation at the southeastern margin of the Congo Craton; zircon U–Pb geochronology of plutonic, volcanic and clastic units in northern Zambia. *Precambrian Res.* **159**, 95-116 (2007).

50. Hanson, R. E. Proterozoic geochronology and tectonic evolution of southern Africa. *Geol. Soc. London* **206**, 427-463 (2003).

51. Kokonyangi, J. et al. U–Pb zircon geochronology and petrology of granitoids from Mitwaba (Katanga, Congo): implications for the evolution of the Mesoproterozoic Kibaran belt. *Precambrian Res.* **132**, 79-106 (2004).

52. Tack, L. et al. The 1375 Ma "Kibaran event" in Central Africa: Prominent emplacement of bimodal magmatism under extensional regime. *Precambrian Res.* **180**, 63-84 (2010).

53. Grantham, G. H. et al. in: *Proterozoic East Gondwana: Supercontinent Assembly and Breakup* (eds Yoshida, M., Windley, B. F. & Dasgupta, S.) 401-425 (Geol. Soc. London Spec. Publ., 2003).

54. Johnson, P. R. & Woldehaimanot, B. Development of the Arabian-Nubian Shield: perspectives on accretion and deformation in the northern East African Orogen and the assembly of Gondwana. *Geol. Soc. London* **206**, 289-325 (2003).

55. Kieffer, B. et al. Flood and shield basalts from Ethiopia: magmas from the African superswell. *J. Petrol.* **45**, 793-834 (2004).

56. Thiéblemont, D. et al. Geological map of Africa at 1:10M scale. Geol. Map. CGMW-BRGM (2016).

57. Gärtner, A. et al. Implications for sedimentary transport processes in southwestern Africa: a combined zircon morphology and age study including extensive geochronology databases. *Int. J. Earth. Sci.* **111**, 767-788 (2022).

58. Wriston, T. A. *The Late Stone Age to Early Iron Age in Hwange National Park, Zimbabwe: Using Archaeology, Soils, Sediments, and Stable Isotopes to Trace Past Peoples and Environments*. Doctoral Thesis (University of Nevada, Nevada, 2013).

59. Soderberg, K. & Compton, J. S. Dust as a nutrient source for fynbos ecosystems, South Africa. *Ecosystems* **10**, 550-561 (2007).

60. Scott, M. et al. Lead and strontium isotopes as palaeodietary indicators in the Western Cape of South Africa. *South Afr. J. Sci.* **116**, 1-8 (2020).

61. Balasse, M. et al. The seasonal mobility model for prehistoric herders in the south-western Cape of South Africa assessed by isotopic analysis of sheep tooth enamel. *J. Archaeol. Sci.* **29**, 917-932 (2002).

62. Stewart, B. A. et al. Ostrich eggshell bead strontium isotopes reveal persistent macroscale social networking across late Quaternary southern Africa. *Proc. Natl Acad. Sci. USA* **117**, 6453-6462 (2020).

63. Cerling, T. E. et al. Stable isotope ecology of black rhinos (*Diceros bicornis*) in Kenya. *Oecologia* **187**, 1095-1105 (2018).

64. Dominy, N. J. et al. Mummified baboons reveal the far reach of early Egyptian mariners. *eLife* **9**, e60860 (2020).

65. Arnold, E. R. et al. Domestic cattle mobility in early farming villages in southern Africa: harvest profiles and strontium ($^{87}Sr/^{86}Sr$) isotope analyses from Early Iron Age sites in the lower Thukela River Valley of South Africa. *Archaeol. Anthropol. Sci.* **5**, 129-144 (2013).

66. House, M. et al. Investigating cattle procurement at Great Zimbabwe using $^{87}Sr/^{86}Sr$. *J. Afr. Archaeol.* **19**, 146-158 (2021).

67. Vorster, C. et al. The determination of $^{11}B/^{10}B$ and $^{87}Sr/^{86}Sr$ isotope ratios by quadrupole-based ICP-MS for the fingerprinting of South African wine. *South Afr. J. Chem.* **63**, 207-214 (2010).

68. Lehmann, S. B. et al. Environmental and ecological implications of strontium isotope ratios in mid-Pleistocene fossil teeth from Elandsfontein, South Africa. *Palaeogeogr. Palaeoclimatol. Palaeoecol.* **490**, 84-94 (2018).

69. Sealy, J. et al. Beyond lifetime averages: tracing life histories through isotopic analysis of different calcified tissues from archaeological human skeletons. *Antiquity* **69**, 290-300 (1995).

70. Balter, V. et al. Evidence for dietary change but not landscape use in South African early hominins. *Nature* **489**, 558-560 (2012).

71. Hamilton, M. I.. *Reconstructing Landscape Use Patterns Using Strontium Isotope Ratios. Doctoral Thesis* (The University of New Mexico, Albuquerque, New Mexico, 2018).

72. Rodrigues, C. et al. Strontium and oxygen isotope fingerprinting of green coffee beans and its potential to proof authenticity of coffee. *Eur. Food. Res. Technol.* **232**, 361-373 (2011).

73. Hamilton, M. I. et al. Using strontium isotopes to determine philopatry and dispersal in primates: a case study from Kibale National Park. *R. Soc. Open Sci.* **8**, 200760 (2021).

74. Talbot, M. R. et al. Strontium isotope evidence for late Pleistocene reestablishment of an integrated Nile drainage network. *Geology* **28**, 343-346 (2000).

75. van der Lubbe, H. J. L. et al. Gradual or abrupt? Changes in water source of Lake Turkana (Kenya) during the African Humid Period inferred from Sr isotope ratios. *Quat. Sci. Rev.* **174**, 1-12 (2017).

76. Coutu, A. N. et al. Mapping the elephants of the 19th century East African ivory trade with a multi-isotope approach. *PLOS ONE* **11**, e0163606 (2016).

77. Copeland, S. R. et al. Using strontium isotopes to study site accumulation processes. *J. Taphon.* **8**, 115-127 (2010).

78. Koch, P. L. et al. Isotopic tracking of change in diet and habitat use in African elephants. *Science* **267**, 1340-1343 (1995).

79. Radloff, F. G. T. et al. Strontium isotope analyses of large herbivore habitat use in the Cape Fynbos region of South Africa. *Oecologia* **164**, 567-578 (2010).

80. Sealy, J. C. et al. $^{87}Sr/^{86}Sr$ as a dietary indicator in modern and archaeological bone. *J. Archaeol. Sci.* **18**, 399-416 (1991).

81. Vogel, J. C. et al. Isotope fingerprints in elephant bone and ivory. *Nature* **346**, 747-749 (1990).

82. Tucker, L. et al. Initial assessment of bioavailable strontium at Oldupai Gorge, Tanzania: Potential for early mobility studies. *J. Archaeol. Sci.* **114**, 105066 (2020).

83. Hamilton, M. et al. Detecting riparian habitat preferences in "savanna" chimpanzees and associated Fauna with strontium isotope ratios: Implications for reconstructing habitat use by the chimpanzee-human last common ancestor. *Am. J. Phys. Anthropol.* **170**, 551-564 (2019).

84. Pye, K. Isotope and trace element analysis of human teeth and bones for forensic purposes. *Geol. Soc. Lond. Spec. Publ.* **232**, 215-236 (2004).

85. Copeland, S. R. et al. Strontium isotope evidence for landscape use by early hominins. *Nature* **474**, 76-78 (2011).

86. Copeland, S. R. et al. in: *Hofmeyr: A Late Pleistocene Human Skull from South Africa* (ed Grine, F. E.) 47-68 (Springer-Cham, 2023).

87. Fowler, K. D. et al. The provisioning of nineteenth century Zulu capitals, South Africa: Insights from strontium isotope analysis of cattle remains. *J. Archaeol. Sci. Rep.* **31**, 102306 (2020).

88. Van der Merwe, N. J. et al. Source-area determination of elephant ivory by isotopic analysis. *Nature* **346**, 744-746 (1990).

89. Hartmann, J. & Moosdorf, N. The new global lithological map database GLiM: A representation of rock properties at the Earth surface. *Geochem. Geophy. Geosyst.* **13**, Q12004 (2012).

90. Zomer, R. J. et al. Climate change mitigation: A spatial analysis of global land suitability for clean development mechanism afforestation and reforestation. *Agr. Ecosyst. & Environ.* **126**, 67-80 (2008).

91. Mooney, W. D. et al. CRUST 5.1: A global crustal model at 5° × 5°. *J. Geophys. Res.* **103**, 727-747 (1998).

92. Vet, R. et al. A global assessment of precipitation chemistry and deposition of sulfur, nitrogen, sea salt, base cations, organic acids, acidity and pH, and phosphorus. *Atmos. Environ.* **93**, 3-100 (2014).

93. Mahowald, N. M. et al. Change in atmospheric mineral aerosols in response to climate: Last glacial period, preindustrial, modern, and doubled carbon dioxide climates. *J. Geophys. Res.* **111**, D10202 (2006).

94. Jarvis, A. et al. Hole-filled SRTM for the globe Version 4. CGIAR-CSI SRTM 90m Database. https://srtm.csi.cgiar.or (2008).

95. Hengl, T. et al. SoilGrids250m: Global gridded soil information based on machine learning. *PLOS ONE* **12**, e0169748 (2017).

96. Balmino, G. et al. Spherical harmonic modelling to ultra-high degree of Bouguer and isostatic anomalies. *J. Geod.* **86**, 499-520 (2012).

97. Hijmans, R. J. et al. Very high resolution interpolated climate surfaces for global land areas. *Int. J. Climatol.* **25**, 1965-1978 (2005).

98. Lu, C. & Tian, H. Global nitrogen and `phosphorus fertilizer use for agriculture production in the past half century: shifted hot spots and nutrient imbalance. *Earth Syst. Sci. Data* **9**, 181-192 (2017).

99. Fleskes, R. E. et al. Ancestry, health, and lived experiences of enslaved Africans in 18th century Charleston: An osteobiographical analysis. *Am. J. Phys. Anthropol.* **175**, 3-24 (2021).

100. Bastos, M. Q. R. et al. Isotopic study of geographic origins and diet of enslaved Africans buried in two Brazilian cemeteries. *J. Archaeol. Sci.* **70**, 82-90 (2016).
