# Supplementary material for: Strontium isoscape of sub-Saharan Africa allows tracing origins of victims of the transatlantic slave trade
Source: Nat Commun. 2024 Dec 30;15:10891. doi: 10.1038/s41467-024-55256-0 (PMC11685951; doi:10.1038/s41467-024-55256-0)
Supplement: Supplementary file 3 — Description of Additional Supplementary Files [file 41467_2024_55256_MOESM3_ESM.pdf]

Description of Additional Supplementary Information for

Strontium isoscape of sub-Saharan Africa allows tracing origins of victims of the transatlantic slave trade

Legends for Supplementary Data 1, Supplementary Data 2, Supplementary Data 3, and Supplementary Data 4:

Supplementary Data 1. The dataset of bioavailable strontium isotopes in sub-Saharan Africa.

Supplementary Data 2. List of geological, climatic, topographic and environmental variables used in the model. P = Polygon; R = Raster.

Supplementary Data 3. Previously published strontium isotope data of human tooth enamel from the Anson Street African Burial Ground in Charleston (USA).

Supplementary Data 4. Previously published isotope data of human tooth enamel from the Pretos Novos Cemetery, Rio de Janeiro (Brazil).
