# Supplementary material for: Strontium isoscape of sub-Saharan Africa allows tracing origins of victims of the transatlantic slave trade
Source: Nat Commun. 2024 Dec 30;15:10891. doi: 10.1038/s41467-024-55256-0 (PMC11685951; doi:10.1038/s41467-024-55256-0)
Supplement: Supplementary file 8 — Reporting Summary [file 41467_2024_55256_MOESM8_ESM.pdf]

nature portfolio

Corresponding author(s): Vicky M. Oelze

Last updated by author(s): Nov 5, 2024

Reporting Summary

Nature Portfolio wishes to improve the reproducibility of the work that we publish. This form provides structure for consistency and transparency in reporting. For further information on Nature Portfolio policies, see our Editorial Policies and the Editorial Policy Checklist.

Statistics

For all statistical analyses, confirm that the following items are present in the figure legend, table legend, main text, or Methods section.

n/a	Confirmed	
[ ]	[x]	The exact sample size (*n*) for each experimental group/condition, given as a discrete number and unit of measurement
[ ]	[x]	A statement on whether measurements were taken from distinct samples or whether the same sample was measured repeatedly
[x]	[ ]	The statistical test(s) used AND whether they are one- or two-sided<br>*Only common tests should be described solely by name; describe more complex techniques in the Methods section.*
[ ]	[x]	A description of all covariates tested
[x]	[ ]	A description of any assumptions or corrections, such as tests of normality and adjustment for multiple comparisons
[ ]	[x]	A full description of the statistical parameters including central tendency (e.g. means) or other basic estimates (e.g. regression coefficient) AND variation (e.g. standard deviation) or associated estimates of uncertainty (e.g. confidence intervals)
[x]	[ ]	For null hypothesis testing, the test statistic (e.g. *F*, *t*, *r*) with confidence intervals, effect sizes, degrees of freedom and *P* value noted<br>*Give P values as exact values whenever suitable.*
[x]	[ ]	For Bayesian analysis, information on the choice of priors and Markov chain Monte Carlo settings
[x]	[ ]	For hierarchical and complex designs, identification of the appropriate level for tests and full reporting of outcomes
[ ]	[x]	Estimates of effect sizes (e.g. Cohen's *d*, Pearson's *r*), indicating how they were calculated

Our web collection on statistics for biologists contains articles on many of the points above.

Software and code

Policy information about availability of computer code

Data collection	n/a
Data analysis	We used ArcGIS Desktop 10.8 for Figure 1 and Supplementary Figure 4, and OriginPro 2021 for Figure. 3a, Figure. 4a, Supplementary Figures 5 and 6. All other analyses and figures were conducted in the R environment, utilizing various packages for specific tasks, including data handling and visualization (tidyverse v. 1.3.1), spatial data management (sf v. 1.0-6; terra v. 1.5-17; rnaturalearth v. 0.1.0), variable selection (VSURF v. 1.1.0; spatialRF v. 1.1.3), random forest regressions (ranger v. 0.13.1; caret v. 6.0-92; tuneRanger v. 0.5), mobility-oriented parity (mop v. 0.1.1), spline correlograms (ncf v. 1.3-2), partial dependence plots (pdp v. 0.7.0), and geographic origin inference (assignR v. 2.1.1).

For manuscripts utilizing custom algorithms or software that are central to the research but not yet described in published literature, software must be made available to editors and reviewers. We strongly encourage code deposition in a community repository (e.g. GitHub). See the Nature Portfolio guidelines for submitting code & software for further information.

Data

Policy information about availability of data

All manuscripts must include a data availability statement. This statement should provide the following information, where applicable:

- Accession codes, unique identifiers, or web links for publicly available datasets
- A description of any restrictions on data availability
- For clinical datasets or third party data, please ensure that the statement adheres to our policy

The raw data supporting the findings of this study, source data, and methods are fully available within the article and its Supplementary Information. The R scripts

used for data analysis, all raster files for modeling and the resulting isoscapes can be found in the Figshare with the following link https://doi.org/10.6084/m9.figshare.23118212.v1.

Research involving human participants, their data, or biological material

Policy information about studies with human participants or human data. See also policy information about sex, gender (identity/presentation), and sexual orientation and race, ethnicity and racism.

Reporting on sex and gender	Our study does not involve human participants
Reporting on race, ethnicity, or other socially relevant groupings	Our study does not involve human participants
Population characteristics	n/a
Recruitment	n/a
Ethics oversight	No ethics oversight was required in this study

Note that full information on the approval of the study protocol must also be provided in the manuscript.

Field-specific reporting

Please select the one below that is the best fit for your research. If you are not sure, read the appropriate sections before making your selection.

☐ Life sciences ☒ Behavioural & social sciences ☐ Ecological, evolutionary & environmental sciences

For a reference copy of the document with all sections, see nature.com/documents/nr-reporting-summary-flat.pdf

Behavioural & social sciences study design

All studies must disclose on these points even when the disclosure is negative.

Study description	We have developed the strontium isotope landscape (isoscape) for sub-Saharan Africa, using quantitative data from an extensive collection of new isotope measurements combined with previously published data. This was achieved through a machine learning modeling approach.
Research sample	We obtained and analyzed 778 environmental samples, including modern plants, soils, and archaeological/modern microfauna, which were collected from a variety of geographic locations across sub-Saharan Africa. These samples represent diverse geological, ecological, and climatic conditions. Additionally, we combined these new data with 1488 previously published Sr isotope datasets. This combination enhances the representativeness of our study and provides a more comprehensive analysis of environmental bioavailable Sr isotopic patterns across the region.
Sampling strategy	Sampling focused on not yet represented regions of sub-Saharan Africa, particularly West and western central Africa. Most environmental samples were obtained within the scope of the Pan African Programme: The Cultured Chimpanzee, which included 38 remote field sites and two nationwide surveys (Liberia and Equatorial Guinea). Other samples (archaeological microfauna/soils) were collected from archaeological fieldwork. All samples were collected in locations avoiding potential anthropogenic Sr sources such as farming fields and roads (fertilizers, pesticides, and traffic pollutants) by at least 500 meters. In most cases, we analyzed 2-4 samples from the same site/location to capture natural variability in Sr isotope ratios. We combined these samples to all of the existing published Sr isotopic data for sub-Saharan African and list all sources in Supplementary Materials. The resulting dataset (n = 2266) represents by far the largest Sr isotopic study of Africa, which spans 35 African countries.
Data collection	All samples (n = 608) were shipped to the University of California, Santa Cruz, Arizona State University (n = 155), and the Max Planck Institute for Evolutionary Anthropology (n = 15) for Sr isotope analysis. At UCSC, Sr isotopes were measured using an IsotopX Phoenix X62 Thermal Ionization Mass Spectrometer (TIMS) in the W.M. Keck Isotope Laboratory, conducted by Xueye Wang, Vicky M. Oelze, and Renée Boucher. At Arizona State University, Sr isotopes were measured using a Thermo-Fisher Neptune Multi Collector-Inductively Coupled Plasma-Mass Spectrometer (MC-ICP-MS) by Andrew M. Zipkin. At the Max Planck Institute for Evolutionary Anthropology, Sr isotope analysis was performed by Sandi R. Copeland. The international NIST SRM-987 standard was run concurrently with the samples in all three labs, with results consistent with the SRM-987 standard value of 0.710250. Further details are provided in the Methods section.
Timing	The samples used in this study were continuously collected during fieldwork conducted between 2012 and 2022.
Data exclusions	No data were excluded from our analyses
Non-participation	n/a
Randomization	n/a

Reporting for specific materials, systems and methods

We require information from authors about some types of materials, experimental systems and methods used in many studies. Here, indicate whether each material, system or method listed is relevant to your study. If you are not sure if a list item applies to your research, read the appropriate section before selecting a response.

Materials & experimental systems

n/a	Involved in the study
☒	☐ Antibodies
☒	☐ Eukaryotic cell lines
☐	☒ Palaeontology and archaeology
☐	☒ Animals and other organisms
☒	☐ Clinical data
☒	☐ Dual use research of concern
☐	☒ Plants

Methods

n/a	Involved in the study
☒	☐ ChIP-seq
☒	☐ Flow cytometry
☒	☐ MRI-based neuroimaging

Palaeontology and Archaeology

Specimen provenance	The archaeological faunal samples analyzed in this study include 1-2g bone and tooth fragments from two rodents collected in Senegal, three specimens (turtle, rodents) from The Gambia, and 18 specimens (bivalve, dog, rodents, bovid) from northern Benin. All samples were obtained during excavations, with the necessary permissions granted by the respective authorities in each country. These permissions and the relevant details are listed in the Supplementary Note 4 of our study.
Specimen deposition	Sampling method is destructive, and small amounts of archaeological samples have been acid digested. All remaining environmental samples are stored at the University of California, Santa Cruz (Santa Cruz, CA, USA), and can be accessed upon request by contacting Vicky M. Oelze at voelze@ucsc.edu.
Dating methods	The chronologies of these archaeological samples are irrelevant for this study.

☒ Tick this box to confirm that the raw and calibrated dates are available in the paper or in Supplementary Information.

Ethics oversight	No ethics oversight was required in this study.

Note that full information on the approval of the study protocol must also be provided in the manuscript.

Animals and other research organisms

Policy information about studies involving animals; ARRIVE guidelines recommended for reporting animal research, and Sex and Gender in Research

Laboratory animals	n/a
Wild animals	Snail shells analyzed in these study are from dead land snails of various species collected opportunistically. No living snail was harmed in this study.
Reporting on sex	n/a
Field-collected samples	Snail shells analyzed in these study are from dead land snails of various species collected opportunistically. No special conditions were required for storing or handing snail shell fragments.
Ethics oversight	n/a

Note that full information on the approval of the study protocol must also be provided in the manuscript.

Dual use research of concern

Policy information about dual use research of concern

Hazards

Could the accidental, deliberate or reckless misuse of agents or technologies generated in the work, or the application of information presented in the manuscript, pose a threat to:

No	Yes	
☒	☐	Public health
☒	☐	National security
☒	☐	Crops and/or livestock
☒	☐	Ecosystems
☒	☐	Any other significant area

Experiments of concern

Does the work involve any of these experiments of concern:

No	Yes	
☒	☐	Demonstrate how to render a vaccine ineffective
☒	☐	Confer resistance to therapeutically useful antibiotics or antiviral agents
☒	☐	Enhance the virulence of a pathogen or render a nonpathogen virulent
☒	☐	Increase transmissibility of a pathogen
☒	☐	Alter the host range of a pathogen
☒	☐	Enable evasion of diagnostic/detection modalities
☒	☐	Enable the weaponization of a biological agent or toxin
☒	☐	Any other potentially harmful combination of experiments and agents

Plants

Seed stocks

Our dataset comprises sampling of 419 wild, non-protected plants from the field collected over the past ten years in mostly remote areas of sub-Saharan Africa. These samples were collected in locations avoiding potential anthropogenic Sr sources such as farming fields and roads (fertilizers, pesticides, and traffic pollutants) by at least 500 meters.

Novel plant genotypes

n/a

Authentication

n/a
